# Extract from Sea Buckthorn Seeds—A Phytochemical, Antioxidant, and Hemostasis Study; Effect of Thermal Processing on Its Chemical Content and Biological Activity In Vitro

**DOI:** 10.3390/nu15030686

**Published:** 2023-01-29

**Authors:** Natalia Sławińska, Jerzy Żuchowski, Anna Stochmal, Beata Olas

**Affiliations:** 1 Department of General Biochemistry, Faculty of Biology and Environmental Protection, University of Lodz, 90-236 Lodz, Poland; 2 Department of Biochemistry and Crop Quality, Institute of Soil Science and Plant Cultivation, State Research Institute, 24-100 Pulawy, Poland

**Keywords:** antioxidant, coagulation, phytochemicals, phenolic compounds, sea buckthorn, thermal processing

## Abstract

Sea buckthorn (*Hippophae rhamnoides* L.) is a small tree, valued for its medicinal properties throughout the ages. Sea buckthorn berries and leaves are a known source of phytochemicals and have been used in the treatment of inflammation, oedema, hypertension, ulcers, and wounds in folk medicine. Sea buckthorn seeds are natural dietary sources of various bioactive compounds as well, but the number of studies on their content and biological properties is still insufficient. For the first time, we examined the phytochemical content and biological activity of sea buckthorn seeds in vitro. We have studied the effect of two extracts—from regular (no thermal processing) and roasted (thermally processed) sea buckthorn seeds—on the levels of oxidative stress induced by H_2_O_2_/Fe^2+^ in plasma, coagulation times, and white thrombus formation (measured by Total Thrombus-formation Analysis System—T-TAS). We observed that sea buckthorn seeds contain diverse flavonoids, mostly glycosides of isorhamnetin, kaempferol, and quercetin, as well as smaller amounts of proanthocyanidins and catechin, triterpenoid saponins, and a number of unidentified polar and hydrophobic compounds. Both extracts inhibited lipid peroxidation and protein carbonylation, but only the extract from roasted seeds decreased oxidation of thiol groups in plasma treated with H_2_O_2_/Fe^2+^. They did not alter coagulation times, but the extract from roasted seeds at the highest concentration (50 µg/mL) prolonged the time needed for white thrombus formation. The results indicate that sea buckthorn seeds have antioxidant activity that is not impaired by thermal processing and possess anticoagulant potential, but more research is needed in order to ascertain which compounds are responsible for these effects, especially in in vivo models.

## 1. Introduction

Sea buckthorn (*Hippophae rhamnoides* L.) is a small tree belonging to the *Elaeagnaceae* (oleaster) family. Not only in vitro, but also in vivo studies indicate that preparations obtained from different parts of sea buckthorn, including fruits and leaves, are sources of various bioactive compounds that have antioxidant, anti-platelet, anticoagulant, anti-tumor, or anti-ulcer activity [1,2,3,4,5]. However, the number of studies examining the biological activity of sea buckthorn seeds in particular is still insufficient [6,7]. Sea buckthorn seeds are rich in various unsaturated fatty acids (40% linoleic, 20% linolenic, and 17% oleic acid), and are a source of phenolic compounds [7,8,9]. Arimboor et al. have reported, that seed kernels had higher content of phenolic acids than seed coat and leaves [9]. In a study by Zhao et al., HPLC analysis showed that the seeds contained protocatechuic acid (0.1278 ± 0.002 mg/g), rutin (0.134 ± 0.002 mg/g), quercetin (0.4128 ± 0.005 mg/g), kaempferol (0.787 ± 0.003 mg/g), and isorhamnetin (0.5509 ± 0.006 mg/g) [10]. 

Antioxidant activity of sea buckthorn seeds has been described only in several papers [7,11,12,13]. For example, Fan et al. have reported that various seed extracts had good DPPH radical scavenging capacity, which was positively correlated with the content of proanthocyanidins [7]. 

Evidence shows that phenolic compounds play an important role in protection of human organs from oxidative stress and inflammatory, neurodegenerative, or cardiovascular diseases (CVDs). Sea buckthorn seeds are not as well-known in phytopharmacology as its other organs (e.g., fruits). Therefore, in continuation of our previous study, we aimed to determine the phytochemical content of sea buckthorn seeds and assess their biological properties related to oxidative stress and hemostasis in vitro. Biological activity was determined in human plasma according to selected biomarkers of oxidative stress and coagulation parameters. In addition, the anticoagulant potential was determined in whole blood, using the Total Thrombus-formation Analysis System (T-TAS). We also compared biological effects of the extract from sea buckthorn seeds with the activity of phenolic and non-polar extracts from sea buckthorn fruits, as our earlier experiments showed that they possess antioxidant and anticoagulant properties as well [14].

Nowadays, the interest in new nutraceuticals and functional foods that could decrease the occurrence of civilization diseases is rising. Different forms of health-promoting food products based on sea buckthorn fruits or oil are already available in various countries. As sea buckthorn seeds are a rich source of nutrients and contain various phytochemicals beneficial to human health, they may find potential use in functional foods, for example as an additive for bakery products. As in this scenario seeds would be subjected to high temperatures, another aim of this study was to investigate the effect of thermal processing on their chemical content and biological activity (in vitro). It is an important to note that all tested extracts were used within the concentration range 0.5–50 µg/mL, which may correspond to the concentrations obtained in blood during supplementation with phenolic compounds.

## 2. Materials and Methods

### 2.1. Chemicals

All reagents represented analytical grades and were purchased from commercial suppliers (including Merck (Darmstadt, Germany), POCh (Gliwice, Poland), Acros (Poznań, Poland), Kselmed (Grudziądz, Poland), and Chempur (Piekary Śląskie, Poland)).

### 2.2. Plant Material

Whole branches of sea buckthorn (*H. rhamnoides* L.) were obtained from a horticultural farm in Sokółka, Podlaskie Voivodeship, Poland (53°24′ N, 23°30′ E). The fruits were hand-picked and stored in a freezer. To isolate sea buckthorn seeds, the fruit was thawed and homogenized in water with a blender. The seeds sedimented, and the fruit pulp and skins were washed out with tap water. The isolated seeds were shortly submerged (for a few seconds) in a small volume of methanol (to remove excess of water and facilitate drying), drained, and air-dried. The dried seeds were stored at ambient temperature.

A portion of sea-buckthorn seeds was subjected to thermal treatment in the Bakery and Confectionery of Łukasz Skałban, Skowieszyn 75A, 24-130 Końskowola. The temperature of the air-steam mixture in the baking chamber at the beginning of (approx. 10 min) was 280 °C, and the relative humidity was 35%. In the second phase, the temperature was lowered to 175 °C. The total roasting time was 36 min. The conditions were chosen to mirror the process of rye bread baking. Regular and roasted seeds obtained in this way were subjected to qualitative and quantitative tests.

### 2.3. Preparation of Sea Buckthorn Seed Extracts and Fruit Extracts (Phenolic and Non-Polar Extract)

#### 2.3.1. The Extract from Sea Buckthorn Seeds 

Sea buckthorn seeds were milled in a coffee grinder. The powdered seeds were defatted with hexane at ambient temperature and dried. A 282.4 g portion of the defatted plant material was extracted with 2.5 L of 80% methanol (*v*/*v*), for 5 h, at ambient temperature, and then with 2.5 L of methanol, under the same conditions. The filtered extracts were pooled, defatted by liquid-liquid extraction with hexane, and rotary evaporated to remove organic solvents. The residue was diluted to the volume of about 400 mL with Milli-Q water, and subjected to liquid-liquid extraction with butanol (4 × ~100 mL). The butanolic extract was rotary evaporated to dryness, dissolved in 20% *tert*-butanol in Milli-Q water, and freeze-dried (Gamma 2-16 LSC, Christ, Osterode am Harz, Germany), to yield 1.566 g of the seed extract.

#### 2.3.2. The Extract from Roasted Sea Buckthorn Seeds

Roasted sea buckthorn seeds were milled in a coffee grinder, defatted with hexane at ambient temperature, and dried. The dried material (91 g) was subjected to a four-step ultrasound-assisted extraction in an ultrasonic bath: 500 mL 80% methanol (*v*/*v*; 30 min), 400 mL 80% methanol (30 min.), 300 mL 80% methanol (30 min.), and 200 mL 100% methanol (20 min.). The filtered extracts were pooled, defatted by liquid-liquid extraction with hexane, and rotary evaporated to remove organic solvents. Next steps of the extract preparation were the same as described above. Finally, 1.930 g of the extract was obtained.

#### 2.3.3. The Extracts from Sea Buckthorn Fruits

Frozen fruits were ground, lyophilized, and defatted with hexane (Soxhlet extractor). 1835 g of defatted material was extracted twice with 80% methanol (the extraction was enhanced with sonification), filtered, concentrated with a rotary-evaporator, and extracted with *n*-hexane. The defatted extract was concentrated under reduced pressure to remove organic solvents, and the residue was suspended in Milli-Q water. The final volume was approximately 1500 mL. Next, the extract was extracted with n-butanol, rotary-evaporated, suspended in Milli-Q water and freeze-dried. Due to the high content of sugars and organic acids, the extract was further purified by SPE. Finally, the extract was fractionated to obtain the phenolic extract (19.62 g) and the non-polar extract (5.04 g). A more detailed description of this procedure can be found at [14]. 

### 2.4. UHPLC-MS Analyses of Extracts from Sea Buckthorn Seeds

UHPLC-MS analyses of the sea buckthorn seed extracts were performed using an ACQUITY UPLC system (Waters Inc., Milford, MA, USA), equipped with a PDA detector, and a triple quadrupole mass detector (TQD; Waters). Samples were chromatographed on an ACQUITY BEH C18 (100 × 2.1 mm, 1.7 μm; Waters) column, the injection volume was 2.5 µL. Mobile phase was composed from Mill-Q water with 0.1% formic acid in (solvent A) and acetonitrile with 0.1% formic acid (solvent B). Qualitative LC-MS analyses were performed as previously described [15]. Briefly, the elution method (15 min.) started from a 0.5 min. isocratic step (7% B), followed by a 11.4 min. linear gradient 7–80% B. Mass analyses were performed in negative and positive ionization mode. Negative ionization: capillary voltage was 2.8 kV; cone voltage 45 V; source temperature 150 °C, desolvation temperature 450 °C, cone gas (N_2_) flow 100 L h^−1^, desolvation gas flow 900 L h ^−1^, positive ionization: capillary voltage was 3.1 kV, cone voltage 60 V, other settings—as above. Compounds were identified on the basis of their MS and UV spectra, as well as literature data. Quantitative analyses of flavonoids were performed using the following elution program: 0.0–0.5 min., 2% B; 0.5–10.0 min., 2–32% B; 10.0–10.1, 32–95% B; 10.1–12.1 min., 95% B; 12.1–12.2, 95–2%; 12.2–14.2 min., 2% B. The applied MS settings were the same as those for the qualitative analyses.

### 2.5. Phytochemical Characteristic of Sea Buckthorn Fruit Extracts (Phenolic and Non-Polar Extracts)

Analysis of the chemical composition of phenolic and non-polar extracts was carried out with Thermo Ultimate 3000RS (Thermo Fischer Scientific, Waltham, MA, USA) chromatographic system. The description of this analysis can be found in Olas et al. [14].

### 2.6. Preparation of Stock Plant Extracts

Plant extracts were dissolved in 1 mL 50% dimethyl sulfoxide (DMSO). The final concentrations of the extracts in plasma and blood samples were 0.5, 1, 5, 10, and 50 μg/mL (for oxidative stress assays and coagulation time measurements) or 0.5, 5, and 50 μg/mL (for T-TAS). The final concentration of DMSO in samples was under 0.05% (*v*/*v*). Low concentrations of DMSO do not impact the results.

### 2.7. Blood Samples

Whole human blood was collected at “Diagnostyka” blood collection center (Brzechwy 7A, Lodz, Poland). All donors were non-smoking, healthy volunteers, that did not drink alcohol or take medication (antioxidant and anti-platelet supplementation) for two weeks prior to blood collection. Peripheral blood was drawn into tubes with CPDA (citrate/phosphate/dextrose/adenine; 8.5:1; *v*/*v*; blood/CPDA) (for oxidative stress assays and coagulation time measurements) or 3.2% sodium citrate (for T-TAS). The analysis of blood samples was performed according to the guidelines of the Helsinki Declaration for Human Research. Procedures were conducted with the consent of Bioethics Committee at the University of Łódź (11/KBBN-UŁ/I/2019). Blood for the T-TAS assay was used within 2 h after collection. For oxidative stress assays and coagulation times measurements, plasma was separated from whole blood by differential centrifugation (2800× *g*, 20 min, at room temperature). 

### 2.8. Oxidative Stress Assays

#### 2.8.1. Lipid Peroxidation Measurement

To assess the effect of the extracts on lipid peroxidation in human plasma, the levels of thiobarbituric acid-reactive substances (TBARS) were measured. First, human plasma was incubated (37 °C, 30 min) with an oxidative stress inducer (4.7 mM H_2_O_2_/3.8 mM Fe^2+^/2.5 mM EDTA) and different concentrations of the extracts. Next, the samples were mixed with equal volumes of cold 15% (*v*/*v*) trichloroacetic acid in 0.25 M HCl and 0.37% (*v*/*v*) thiobarbituric acid in 0.25 M HCl, and placed in a heating block (100 °C, 15 min). After cooling, the samples were centrifuged (10,000× *g*, 15 min, 18 °C). The absorbance of the supernatant was measured at 535 nm (SPECTROstar Nano Microplate Reader—BMG LABTECH Germany). The levels of TBARS were calculated with a molar extinction coefficient (ε = 156,000 M^−1^ cm^−1^). The results were expressed as nmol TBARS/mL of plasma [16].

#### 2.8.2. Thiol Group Oxidation Measurement

To measure the levels of thiol groups in plasma, a method with Ellman’s reagent—5,5′-dithio-bis-(2-nitrobenzoic acid) (DTNB) was used. First, human plasma was incubated (37 °C, 30 min) with an oxidative stress inducer (4.7 mM H_2_O_2_/3.8 mM Fe^2+^/2.5 mM EDTA) and different concentrations of the extracts. 20 μg of 10% sodium dodecyl sulfate (SDS) and 160 μg of phosphate buffer (pH 8) were added to 20 μg of the samples, and absorbance was measured at 412 nm (SPECTROstar Nano Microplate Reader—BMG LABTECH Germany). Afterward, 16.6 μL of 10 μM DTNB was added (in blank phosphate buffer pH 8 was added instead), and the samples were incubated at 37 °C for 60 min. Again, absorbance was measured at 412 nm. The content of plasma protein in the samples was determined by measuring absorbance at 280 nm. The levels of thiol groups were calculated with a molar extinction coefficient (ε = 13,600 M^−1^ cm^−1^). The results were expressed as nmol thiol groups/mg of plasma protein [16].

#### 2.8.3. Protein Carbonylation Measurement

To measure the levels of carbonyl groups in plasma, a method with 2,4-dinitrophenylhydrazine (DNPH) was used. First, human plasma was incubated (37 °C, 30 min) with an oxidative stress inducer (4.7 mM H_2_O_2_/3.8 mM Fe^2+^/2.5 mM EDTA) and different concentrations of the extracts. The assay was carried out according to the method described in Levine et al. [11] and Bartosz [16,17]. The levels of carbonyl groups were calculated with a molar extinction coefficient (ε = 22,000 M^−1^ cm^−1^). The content of plasma protein in the samples was determined by measuring absorbance at 280 nm. The results are presented as nmol carbonyl groups/mg of plasma protein. The carbonyl group concentration was measured using the SPECTROstar Nano Microplate Reader, BMG Labtech, Germany.

#### 2.8.4. Measurement of Prothrombin Time

Prothrombin time was determined coagulometrically, according to the method described by Malinowska et al. [18]. First, human plasma was incubated (37 °C, 30 min) with different concentrations of the extracts. Next, the samples (50 μL) were incubated again (37 °C, 2 min), and 100 μL of Dia-PT solution (commercial product—Kselmed, Grudziadz, Poland) was added immediately before the measurements. The measurements were carried out on Optic Coagulation Analyser, model K-3002 (Kselmed, Grudziadz, Poland).

#### 2.8.5. Thrombin Time

Thrombin time was determined coagulometrically, according to the method described by Malinowska et al. [18]. First, human plasma was incubated (37 °C, 30 min) with different concentrations of the extracts. Next, the samples (50 μL) were incubated again (37 °C, 1 min), and 100 μL of thrombin was added immediately before the measurements (final concentration—5 U/mL). The measurements were carried out on Optic Coagulation Analyser, model K-3002 (Kselmed, Grudziadz, Poland).

#### 2.8.6. Activated Partial Thromboplastin Time

Activated partial thromboplastin time was determined coagulometrically, according to the method described by Malinowska et al. [18]. First, human plasma was incubated (37 °C, 30 min) with different concentrations of the extracts. Next, the samples (50 μL) were incubated again (37 °C, 3 min) with 50 μL of Dia-PTT solution (Kselmed, Grudziądz, Poland). Finally, 50 μL of Dia-CaCl_2_ solution (Kselmed, Grudziądz, Poland) was added. The measurements were carried out on Optic Coagulation Analyser, model K-3002 (Kselmed, Grudziadz, Poland).

#### 2.8.7. Total Thrombus-Formation Analysis System (T-TAS) (AR-Chip) 

AR-chip of the T-TAS system can measure white thrombus formation process in semi-physiological conditions. All materials and chemicals for the assay were purchased from Bionicum Sp. z.o.o., Poland. Whole blood (495 µL) was incubated with the extracts at final concentrations of 0.5, 5, and 50 μg/mL (30 min, 37 °C). Next, 480 µL of blood was added to 20 μL of CaCTI reagent (0.3M CaCl_2_, 1.25 mg/mL corn-derived trypsin inhibitor). Afterward, 480 μL of blood mixed with CaCTI was added to a reservoir attached to the AR-chip. The results were recorded as AUC_30_ (Area Under the Curve)—area under the flow pressure curve recorded for 30 min after the start of the test. The AUC_30_ depicts the growth, intensity, and stability of thrombus formation. Data were depicted as % of control. Further information about the assay can be found in Hosokawa et al. [19]. 

### 2.9. Statistical Analysis

Statistical analysis was performed with Statistica 10 (StatSoft 13.3, TIBCO Software Inc. Palo Alto, CA, USA). The distribution of data was checked by Shapiro-Wilk test, and the homogeneity of variance by Levene’s test. Differences within and between groups were assessed with ANOVA followed by Tukey’s test, or Kruskall-Wallis test. The results are presented as means ± SD or medians and interquartile ranges. The results were considered significant at *p* ≤ 0.05. Dixon’s Q-test was used to eliminate uncertain data.

## 3. Results

### 3.1. Phytochemical Analysis of the Extracts

**Figure 1:**
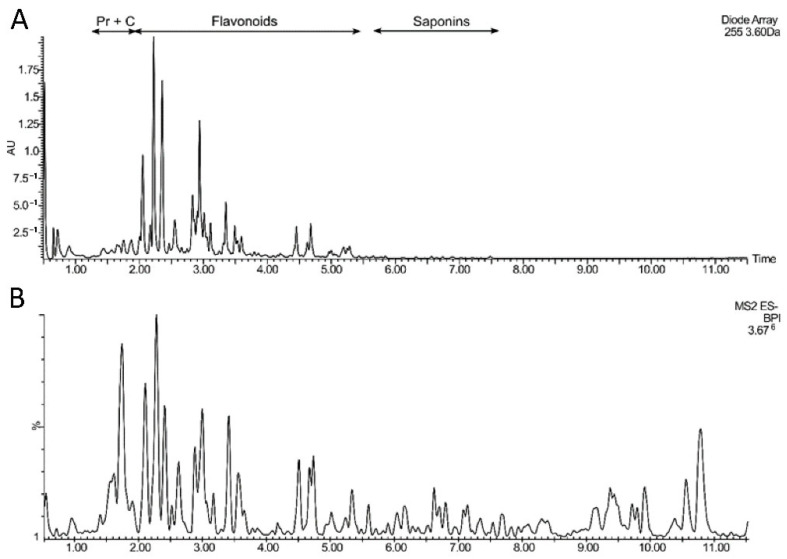
UHPLC-UV (255 nm; (**A**)) and base peak chromatogram (negative ion mode; (**B**)) of sea buckthorn seed extract. Pr + C—proanthocyanidins and catechin.

The composition of the extract from regular sea buckthorn seeds did not differ significantly from the extract from roasted seeds. Both extracts contained diverse flavonoids, glycosides of isorhamnetin, kaempferol, and quercetin (Table 1), with mostly two or three monosaccharide units. Some were acylated with sinapic, ferulic, or menthafolic acid, but they constituted a minor group among the seed flavonoids. Total flavonoid content of regular and roasted seed extracts was 157.2 ± 1.44 mg g^−1^ and 113.5 ± 2.28 mg g^−1^, respectively. Other phenolic compounds were present in smaller amounts and comprised mainly of B-type proanthocyanidins and catechin (Figure 1). Apart from phenolics, the extracts contained triterpenoid saponins which gave deprotonated ions (negative ion mode) at *m*/*z* 1459, 1401, 1297, 1313, 1255, as well as 1269, 1417, 1431. In addition, a number of unidentified polar and hydrophobic compounds were present.

Phenolic extract from sea buckthorn fruits contained mostly glycosides of isorhamnetin and quercetin (including compounds that were acylated with an unidentified aliphatic acid). It contained small amounts of putative and acylated triterpenes as well. The non-polar extract from sea buckthorn fruits contained large amounts of triterpenes (including acylated triterpenes) and other unidentified non-polar compounds. It also contained trace amounts of flavonoid glycosides. A detailed composition of both fruit extracts can be found in Olas et al. [14].

### 3.2. Oxidative Stress Assays

**Figure 2:**
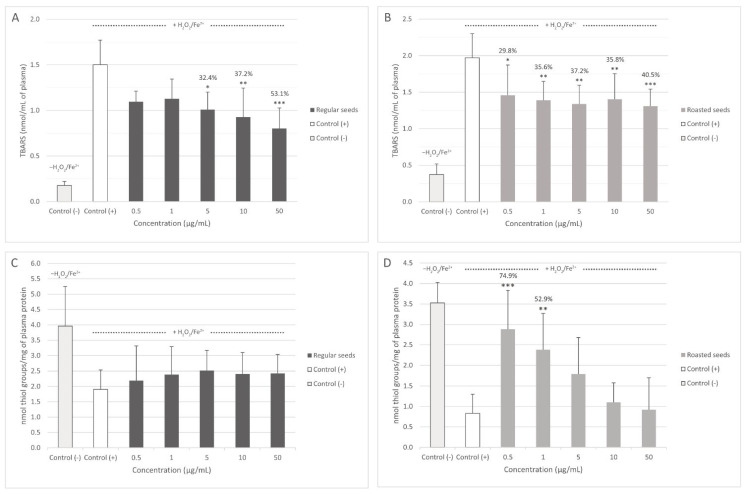
Effect of the extracts from regular (**A**,**C**) and roasted (**B**,**D**) sea buckthorn seeds on the level of thiobarbituric acid reactive substances (TBARS) and on the level of thiol groups in human plasma treated with Fe^2+^/H_2_O_2_ (*n* = 8). Control (+) and test samples (0.5–50 μg/mL) were incubated (30 min, 37 °C) with Fe^2+^/H_2_O_2_—an oxidative stress inducer. The results are presented as nmol TBARS/mL of plasma (**A**,**B**) and the results are also presented as nmol thiol groups/mg of plasma protein (**C**,**D**). The data are expressed as means ± SD. The results were considered significant at *p* < 0.05 (* *p* < 0.05; ** *p* < 0,01; *** *p* < 0.001). The numbers above significant results are % of lipid peroxidation inhibition.

**Figure 3:**
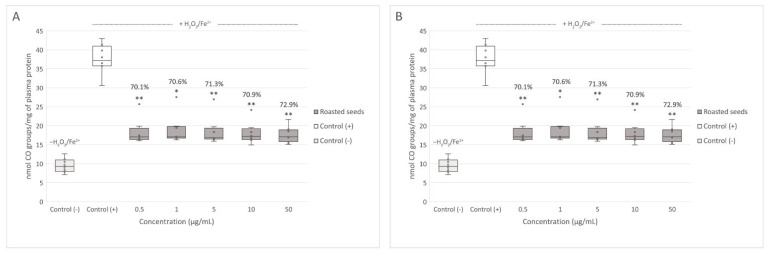
Effect of the extracts from regular (**A**) and roasted (**B**) sea buckthorn seeds (at concentrations 0.5–50 μg/mL) on the level of carbonyl (CO) groups in human plasma treated with Fe^2+^/H_2_O_2_ (*n* = 8). Control (+) and test samples (0.5–50 μg/mL) were incubated (30 min, 37 °C) with Fe^2+^/H_2_O_2_—an oxidative stress inducer. The results are presented as nmol CO groups/mg of plasma protein. The data are expressed as medians and interquartile ranges. The results were considered significant at *p* < 0.05 (* *p* < 0.05; ** *p* < 0,01). The numbers above significant results are % of carbonyl groups oxidation inhibition.

Both extracts inhibited plasma lipid peroxidation induced by H_2_O_2_/Fe^2+^. Regular seed extract significantly decreased lipid peroxidation at concentrations of 5, 10, and 50 μg/mL (Figure 2A), and roasted seed extract at all tested concentrations (0.5–50 μg/mL) (Figure 2B). The differences between the activity of both extracts were not significant. None of the tested concentrations of regular seed extract changed the level of thiol groups in plasma treated with H_2_O_2_/Fe^2+^ (Figure 2C). However, 0.5 and 1 μg/mL of roasted seed extract significantly inhibited thiol group oxidation induced by H_2_O_2_/Fe^2+^ (Figure 2D). Protein carbonylation in plasma treated with H_2_O_2_/Fe^2+^ was inhibited both by the extract from regular (at concentrations of 1–50 μg/mL) (Figure 3A) and roasted seeds (at all tested concentrations (0.5–50 μg/mL) (Figure 3B). In this case, the activity of the extracts was not concentration-dependent. 

### 3.3. Measurement of Coagulation Times (PT, TT, and APTT)

Both tested extracts did not significantly impact coagulation times (PT, TT, and APTT) measured in human plasma at all used concentrations (0.5–50 μg/mL).

### 3.4. Measurement of White Thrombus Formation with T-TAS (AR-Chip)

**Figure 4:**
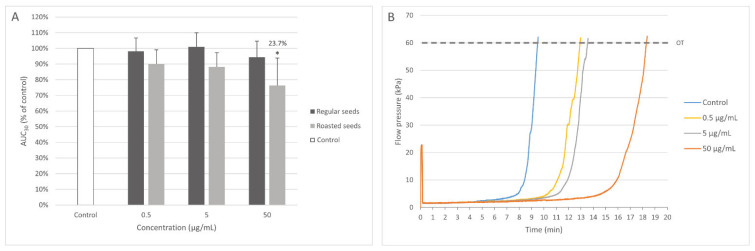
Effect of the extracts from regular and roasted sea buckthorn seeds (at concentrations 0.5, 5, and 50 μg/mL) on white thrombus formation in whole blood (*n* = 5). The samples were analyzed with T-TAS AR-chip, at the shear stress rates of 600/s. The results are presented as AUC_30_ (area under the curve). The data are expressed as means ± SD. The results were considered significant at *p* < 0.05 (* *p* < 0.05). The number above the significant result is % of coagulation inhibition. (**A**). Effect of the extract from roasted sea buckthorn seeds (at concentrations 0.5, 5, and 50 μg/mL) on coagulation in whole blood; OT—occlusion time (**B**). Figure 4B demonstrates selected diagrams (**B**).

Only the extract from roasted seeds at the highest concentration (50 μg/mL) significantly prolonged the time of occlusion, demonstrating anticoagulant activity (Figure 4).

### 3.5. Comparison between Antioxidant Activity of Regular Seed Extract and Phenolic and Non-Polar Extracts from Sea Buckthorn Fruits

**Figure 5:**
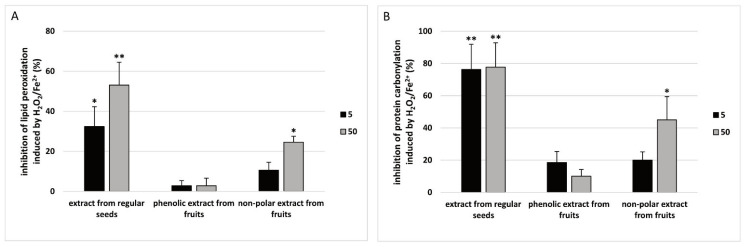
Comparative antioxidant activity of the extract from regular sea buckthorn seeds (at concentrations: 5 and 50 µg/mL, pre-incubation time—5 min), phenolic preparation from sea buckthorn fruits (at concentrations: 5 and 50 µg/mL, pre-incubation time—5 min) and non-polar preparation from sea buckthorn fruits (at concentrations: 5 and 50 µg/mL, pre-incubation time—5 min) in plasma treated with H_2_O_2_/Fe^2+^ (incubation time—25 min). Results are given as mean ± SD (*n* = 8). Kruskal-Wallis test: (**A**) for lipid peroxidation: * *p* < 0.05 (preparation of sea buckthorn seeds (at concentration: 1 µg/mL) vs. phenolic preparation of sea buckthorn fruits (at concentrations: 1 µg/mL) non-polar preparation of sea buckthorn fruits (at concentrations: 5 µg/mL); ** *p* < 0.01 (preparation of sea buckthorn seeds (at concentration: 50 µg/mL) vs. phenolic preparation of sea buckthorn fruits (at concentrations: 50 µg/mL)/non-polar preparation of sea buckthorn fruits (at concentrations: 50 µg/mL); (**B**) for protein carbonylation: * *p* < 0.05, ** *p* < 0.01 (preparation of sea buckthorn seeds (at concentration: 5 and 50 µg/mL) vs. phenolic preparation of sea buckthorn fruits (at concentrations: 5 and 50 µg/mL) non-polar preparation of sea buckthorn fruits (at concentrations: 5 and 50 µg/mL).

It was demonstrated that the extract from regular sea buckthorn seeds (1 and 50 µg/mL) had stronger antioxidant activity (measured by TBARS level (A) and protein carbonylation (B)) than the phenolic extract from sea buckthorn fruits (1 and 50 µg/mL) and the non-polar extract from sea buckthorn fruits (1 and 50 µg/mL) (Figure 5).

## 4. Discussion

Sea buckthorn seed extracts contained diverse flavonoids, mainly non-acylated glycosides of isorhamnetin, kaempferol, and quercetin, most of them with two or three monosaccharides. Similar flavonoids were also reported in earlier studies [20,21]. However, Arimboor and Arumughan [20] detected much higher amounts of isorhamnetin 3-*O*-rutinoside and isorhamnetin 3-*O*-glucoside. The investigated sea buckthorn seed extracts also contained flavonoids acylated with phenolic acids (sinapic, ferulic) or menthafolic acid ((2*E*)-2,6-dimethyl-6-hydroxy-2,7-octadienoic acid), similar to those previously isolated from sea buckthorn seeds and fruits [22,23,24]. Apart from phenolic compounds, the seed extracts contained triterpenoid saponins, mostly the same as those previously detected in sea buckthorn leaves [25], but different from triterpenoid saponins isolated from seeds of *H. rhamnoides* ssp. *sinensis* by Chen et al. [26] and Gao et al. [27].

Cardiovascular diseases (CVDs) can be prevented by modifying various risk factors, such as cholesterol level, blood sugar level, oxidative stress, and body weight. Some papers have suggested that diet rich in fruits and vegetables, and natural preparations with high concentration of phenolic compounds may be a safer alternative for preventing and treating CVDs. There are several studies that describe positive effects of sea buckthorn seeds on the cardiovascular system. For example, in a clinical trial by Vashishtha et al. [13], 0.75 mL of seed oil reduced blood pressure and decreased the levels of total cholesterol, triglycerides, and LDL-cholesterol in both healthy and hypertensive subjects [13]. Chand et al. [28] studied the effect of sea buckthorn seed supplementation (1, 2, and 3 g/kg of feed) on the concentration of cholesterol in eggs. In this experiment, 40 birds that were supplemented sea buckthorn seeds had decreased level of egg cholesterol [28]. Krejcarová et al. [29] also noticed that supplementation of sea buckthorn seeds reduces serum cholesterol concentration [29]. 

Increased probability of thrombotic events is another risk factor in CVDs, especially in ischemic heart disease. Often, patients have to take long-term anticoagulant medication (e.g., aspirin), which can cause adverse effects, therefore new anticoagulant and antiplatelet compounds are still being investigated. Earlier studies have noted anticoagulant activity of preparations from different parts of sea buckthorn, (including fruits, leaves, and twigs) but effects of the seeds on hemostasis have not been investigated yet [25,30]. Here, for the first time we have shown that the extract from roasted seeds at the highest concentration (50 µg/mL) prolonged the time needed for white thrombus formation. These results indicate that sea buckthorn seeds have anticoagulant potential, but more research is needed to ascertain which compounds are responsible for this effect.

Apart from their effects on the cardiovascular system, sea buckthorn seeds have shown other biological activities that might prove beneficial for human health. Zhang et al. [6] studied the anti-melanogenesis activity of hydroalcoholic extract of sea buckthorn seed residues in an in vitro model. The authors identified about 24 compounds in the extract. Kaempferol and its derivatives (2796.2 ± 31.5 µg per g dry weight) were the main compounds. The extract had strong antioxidant properties, with a slight cytotoxic effect on B16F10 mouse melanoma cells. Strong inhibition of extracellular tyrosinase and decreased activity of intracellular tyrosinase were described as well [6]. Wang et al. [11] observed protective properties of proanthocyanidins from sea buckthorn seeds against visible light-induced retinal degeneration (via anti-inflammatory, antioxidant and antiapoptotic mechanisms) in vivo. In this study, pigmented rabbits were supplemented with proanthocyanidins from sea buckthorn seeds (50 and 100 mg/kg/day) for 14 days. Oxidative stress was measured by different parameters, including total antioxidant capacity (T-AOC), the level of malondialdehyde (MDA), and the activity of catalase (CAT) and glutathione peroxidase (GSH-Px) in the retinal homogenate. As the maximum intake of proanthocyanidins in human populations can reach 400–450 mg/day and the average weight of an adult human is considered 60 kg, Wang et al. [11] suggested that the used dose of 50 mg/kg could be considered a moderate dose, while the dose of 100 mg/kg can be considered a high nutritional dose. Biological properties of proanthocyanidins are generally attributed to their metabolites with smaller molecular size, which are formed and absorbed in the colon. Wang et al. [11] suppose that they may be a decisive factor in the protective effect of sea buckthorn proanthocyanidins against oxidative stress [11].

It is important to note, that various technologies used in the food industry are not entirely favorable to active compounds contained in plants. For example, phenolic compounds can be degraded by high temperatures [31]. On the other hand, heat-induced change in the chemical content and structure of phytochemicals can have positive effects as well. For example, Agbaria et al. [32] showed that controlled thermal processing of black seed (*Nigella sativa*) induces significantly higher anti-proliferative activity in mouse colon carcinoma (MC38) in vitro, associated with a higher thymoquinone content in seed oil [32]. Here, we noted some degree of flavonoid loss in thermally processed seeds, but the decrease (157.2 ± 1.44 to 113.5 ± 2.28 mg/g) was small in comparison with a study by Ursache et al. [33], where the content of flavonoids in thermally processed (60–100 °C, 25 min) extracts from sea buckthorn berries was lower by 77.91 to 90.04% [33]. This is consistent with the lack of significant differences in antioxidant activity of the extracts from regular and roasted seeds in regard to lipid peroxidation and protein carbonylation in plasma treated with H_2_O_2_/Fe^2+^.

A comparison between antioxidant activity of the extracts from sea buckthorn seeds and fruits showed, that the regular seed extract was more efficient at preventing lipid peroxidation and protein carbonylation than phenolic and non-polar extracts from the fruits. This is consistent with the results of Sharma et al. [12], who compared the antioxidant activity of various extracts from different parts of sea buckthorn. For example, 2,2′-azino-bis(3-ethylbenzothiazoline-6-sulfonicacid) diammonium salt (ABTS) radical-scavenging ability of the extracts (obtained by microwave-assisted extraction method) from *H. rhamnoides* seeds and fruits were 182.13 ± 0.10 and 18.81 ± 0.19 mg Trolox equivalent/g of plant material, respectively. The results of 2,2′-diphenyl-1-picrylhydrazyl (DPPH) radical scavenging assay indicated higher antioxidant activity of the seeds as well (282.75 ± 0.12 mg Trolox equivalent/g for the seeds and 28.40 ± 0.19 mg Trolox equivalent/g for the fruits). The researchers have also reported, that seed extracts had a higher content of phenolics than fruit extracts, which was correlated with their stronger antioxidant activity [12]. On the other hand, a comparison of antioxidant capacity of different organs of *H. rhamnoides* ssp. *yunnanensis* showed, that the seeds were superior antioxidants only in the Oxygen Radical Absorbance Capacity (ORAC) assay, while the pulp was better at scavenging the ABTS and DPPH radicals [13]. Our previous analysis showed that phenolic extract from sea buckthorn fruits contained mostly glycosides isorhamnetin and quercetin, with small amounts of triterpenes, while non-polar extract contained mostly triterpenes and acylated triterpenes, with only small amounts of flavonoid glycosides [14]. Antioxidant activity of the phenolic extract was the lowest, while the activity of the non-polar extract was lower than the activity of the seed extract, but still evident. Alterations in the profile of flavonoid glycosides in the phenolic extract and seed extract, or in their total flavonoid content might contribute to the differences in their activity. The activity of the non-polar extract indicates that the identified triterpenoids also possess antioxidant potential. A more detailed assessment of the triterpenoid profile of non-polar and seed extracts would show if the compounds they contain are similar.

## 5. Conclusions

Analysis of the chemical content of regular and roasted sea buckthorn seeds showed that they contain diverse flavonoids, mainly non-acylated glycosides of isorhamnetin, kaempferol, and quercetin, similar to compounds previously isolated from sea buckthorn seeds and fruits. Flavonoid loss caused by thermal processing was relatively small in comparison with results of Ursache et al. [33]. For the first time, our data indicate that sea buckthorn seeds possess a wide positive spectrum of biological activity (including not only antioxidant, but also anticoagulant) that is not impaired by thermal processing. This is consistent with other studies describing antioxidant activity of sea buckthorn seeds and anticoagulant activity of other parts of sea buckthorn (including fruits, leaves, and twigs) but more research is needed to ascertain which compounds are responsible for these effects. In depth analysis of the mechanisms responsible for the anticoagulant activity is required as well. In the future, sea buckthorn seeds should also be studied in animal models, to determine if these results can be replicated in vivo.

## Figures and Tables

**Table 1:** Flavonoids in extracts from sea buckthorn seeds.

tR	*m*/*z ^#^*	Tentative Identification	Extract from Sea Buckthorn Seeds (mg g^−1^)	Extract from Sea Buckthorn Roasted Seeds (mg g^−1^)
4.02	771	Q-3-*O*-Hex-Hex-7-*O*-dHex	2.9 ± 0.01 *	2.3 ± 0.13 *
4.10	771	Q-3-*O*-Hex-Hex-7-*O*-dHex	13.5 ± 0.02 *	11.8 ± 0.22 *
4.30	755	K-3-*O*-Hex-Hex-7-*O*-dHex	4.0 ± 0.02 *	2.7 ± 0.07 *
4.40	755	K-3-*O*-Hex-Hex-7-*O*-dHex	25.9 ± 0.17 *	20.7 ± 0.36 *
4.63	785	I-3-*O*-Hex-Hex-7-*O*-dHex	5.3 ± 0.06 *	6.5 ± 0.15 *
4.66	785	I-3-*O*-Hex-Hex-7-*O*-dHex	18.6 ± 0.26 *	18.1 ± 0.28 *
4.97	755	Q-3-*O*-Hex-dHex-7-*O*-dHex	4.8 ± 0.05 *	3.1 ± 0.04 *
5.43	739	K-3-*O*-Hex-dHex-7-*O*-dHex	2.7 ± 0.09 *	1.9 ± 0.04 *
5.55	623	I-3-*O*-Hex-7-*O*-dHex	3.9 ± 0.08 *	2.9 ± 0.06 *
5.71	623	I-3-*O*-Hex-7-*O*-dHex	16.6 ± 0.21 *	10.7 ± 0.19 *
5.80	961	K-Hex-Hex-dHex-Sin	4.4 ± 0.08 *	3.1 ± 0.38 *
5.88	991	I-Hex-Hex-dHex-Sin	5.8 ± 0.08 *	4.7 ± 0.08 *
6.01	961	I-Hex-Hex-Fer-dHex-Fer	2.0 ± 0.03 *	1.3 ± 0.03 *
6.40	623	I-3-*O*-Rut	5.4 ± 0.06	1.6 ± 0.02
8.58	937	quercetin acylated derivative	4.3 ± 0.08 *	1.7 ± 0.02 *
8.91	921	isorhamnetin acylated derivative	2.7 ± 0.02 *	1.1 ± 0.05 *
9.03	951	isorhamnetin acylated derivative	4.3 ± 0.06 *	2.1 ± 0.07 *
		other	30.1 ± 0.39 *	17.2 ± 0.18 *
		Total	157.2 ± 1.44 *	113.5 ± 2.28 *

^#^ negative ion mode; * isorhamnetin 3-O-rutinoside equivalent; I—isorhamnetin; K—kaempferol; Q—quercetin; Hex—hexose; dHex—deoxyhexose; Rut—rutinoside; Fer—ferulic acid; Sin—sinapic acid.

## Data Availability

Not applicable.

## Abbreviations

APTT—activated partial thromboplastin time; CAT—catalase; CVDs—cardiovascular diseases; DMSO—dimethyl sulfoxide; DNPH—2,4-dinitrophenylhydrazine; DTNB—5,5′-dithio-bis-(2-nitrobenzoic acid); GSH-Px—glutathione peroxidase; MDA—malondialdehyde; PT—prothrombin time; T-AOC—total antioxidant capacity; TBARS—thiobarbituric acid-reactive substances; TT—thrombin time; T-TAS—Total Thrombus-formation Analysis System

## References

[1] Suryakumar G., Gupta A. (2011). Medicinal and Therapeutic Potential of Sea Buckthorn (*Hippophae rhamnoides* L.). J. Ethnopharmacol..

[2] Olas B. (2016). Sea Buckthorn as a Source of Important Bioactive Compounds in Cardiovascular Diseases. Food Chem. Toxicol..

[3] Olas B., Skalski B., Ulanowska K. (2018). The Anticancer Activity of Sea Buckthorn [*Elaeagnus rhamnoides* (L.) A. Nelson]. Front. Pharmacol..

[4] Olas B. (2018). The Beneficial Health Aspects of Sea Buckthorn (*Elaeagnus rhamnoides* (L.) A.Nelson) Oil. J. Ethnopharmacol..

[5] Pundir S., Garg P., Dviwedi A., Ali A., Kapoor V.K., Kapoor D., Kulshrestha S., Lal U.R., Negi P. (2021). Ethnomedicinal Uses, Phytochemistry and Dermatological Effects of *Hippophae rhamnoides* L.: A Review. J. Ethnopharmacol..

[6] Zhang J., Wang C., Wang C., Sun B., Qi C. (2018). Understanding the Role of Extracts from Sea Buckthorn Seed Residues in Anti-Melanogenesis Properties on B16F10 Melanoma Cells. Food Funct..

[7] Fan J., Zhang J., Song H., Zhu W., Liu Y. Antioxidant Activity and Phenolic Components of Sea Buckthorn (*Hippophae rhamnoides*) Seed Extracts. Proceedings of the IEEE 2013 International Conference on Advanced Mechatronic Systems.

[8] Cenkowski S., Yakimishen R., Przybylski R., Muir W.E. (2006). Quality of Extracted Sea Buckthorn Seed and Pulp Oil. Can. Biosyst. Eng..

[9] Arimboor R., Kumar K.S., Arumughan C. (2008). Simultaneous Estimation of Phenolic Acids in Sea Buckthorn (*Hippophaë rhamnoides*) Using RP-HPLC with DAD. J. Pharm. Biomed. Anal..

[10] Zhao L., Wen E., Upur H., Tian S. (2017). High Performance Liquid Chromatography-Diode Array Detector Method for the Simultaneous Determination of Five Compounds in the Pulp and Seed of Sea Buckthorn. Pharmacogn. Mag..

[11] Wang Y., Zhao L., Huo Y., Zhou F., Wu W., Lu F., Yang X., Guo X., Chen P., Deng Q. (2016). Protective Effect of Proanthocyanidins from Sea Buckthorn (*Hippophae rhamnoides* L.) Seed against Visible Light-Induced Retinal Degeneration in Vivo. Nutrients.

[12] Sharma U.K., Sharma K., Sharma N., Sharma A., Singh H.P., Sinha A.K. (2008). Microwave-Assisted Efficient Extraction of Different Parts of *Hippophae rhamnoides* for the Comparative Evaluation of Antioxidant Activity and Quantification of Its Phenolic Constituents by Reverse-Phase High-Performance Liquid Chromatography (RP-HPLC). J. Agric. Food Chem..

[13] Vashishtha V., Barhwal K., Kumar A., Hota S.K., Chaurasia O.P., Kumar B. (2017). Effect of Seabuckthorn Seed Oil in Reducing Cardiovascular Risk Factors: A Longitudinal Controlled Trial on Hypertensive Subjects. Clin. Nutr..

[14] Olas B., Żuchowski J., Lis B., Skalski B., Kontek B., Grabarczyk Ł., Stochmal A. (2018). Comparative Chemical Composition, Antioxidant and Anticoagulant Properties of Phenolic Fraction (a Rich in Non-Acylated and Acylated Flavonoids and Non-Polar Compounds) and Non-Polar Fraction from *Elaeagnus rhamnoides* (L.) A. Nelson Fruits. Food Chem..

[15] Żuchowski J., Pecio Ł., Marciniak B., Kontek R., Stochmal A. (2019). Unusual Isovalerylated Flavonoids from the Fruit of Sea Buckthorn (*Elaeagnus rhamnoides*) Grown in Sokółka, Poland. Phytochemistry.

[16] Bartosz G. (2018). Druga Twarz Tlenu.

[17] Levine R.L., Garland D., Oliver C.N., Amici A., Climent I., Lenz A.G., Ahn B.W., Shaltiel S., Stadtman E.R. (1990). Determination of Carbonyl Content in Oxidatively Modified Proteins. Methods Enzymol..

[18] Malinowska J., Kołodziejczyk-Czepas J., Moniuszko-Szajwaj B., Kowalska I., Oleszek W., Stochmal A., Olas B. (2012). Phenolic Fractions from Trifolium Pallidum and Trifolium Scabrum Aerial Parts in Human Plasma Protect against Changes Induced by Hyperhomocysteinemia in Vitro. Food Chem. Toxicol..

[19] Hosokawa K., Ohnishi T., Kondo T., Fukasawa M., Koide T., Maruyama I., Tanaka K.A. (2011). A Novel Automated Microchip Flow-Chamber System to Quantitatively Evaluate Thrombus Formation and Antithrombotic Agents under Blood Flow Conditions. J. Thromb. Haemost..

[20] Arimboor R., Arumughan C. (2012). HPLC-DAD-MS/MS Profiling of Antioxidant Flavonoid Glycosides in Sea Buckthorn (*Hippophae Rhamnoides* L.) Seeds. Int. J. Food Sci. Nutr..

[21] Tkacz K., Wojdyło A., Turkiewicz I.P., Nowicka P. (2021). Triterpenoids, Phenolic Compounds, Macro- and Microelements in Anatomical Parts of Sea Buckthorn (*Hippophaë rhamnoides* L.) Berries, Branches and Leaves. J. Food Compos. Anal..

[22] Li R., Wang Q., Zhao M., Yang P., Hu X., Ouyang D. (2019). Flavonoid Glycosides from Seeds of *Hippophae rhamnoides* Subsp. Sinensis with α-Glucosidase Inhibition Activity. Fitoterapia.

[23] Gao W., Chen C., Kong D.Y. (2013). Hippophins C-F, Four New Flavonoids, Acylated with One Monoterpenic Acid from the Seed Residue of *Hippophae rhamnoides* Subsp. Sinensis. J. Asian Nat. Prod. Res..

[24] Fang R., Veitch N.C., Kite G.C., Porter E.A., Simmonds M.S.J. (2013). Enhanced Profiling of Flavonol Glycosides in the Fruits of Sea Buckthorn (*Hippophae rhamnoides*). J. Agric. Food Chem..

[25] Skalski B., Kontek B., Olas B., Zuchowski J., Stochmal A. (2018). Phenolic Fraction and Nonpolar Fraction from Sea Buckthorn Leaves and Twigs: Chemical Profile and Biological Activity. Future Med. Chem..

[26] Chen C., Gao W., Cheng L., Shao Y., Kong D.Y. (2014). Four New Triterpenoid Glycosides from the Seed Residue of *Hippophae rhamnoides* Subsp. Sinensis. J. Asian Nat. Prod. Res..

[27] Gao W., Chen C., Zhang J., Cheng L., Kong D.Y. (2015). Two New Triterpene Saponins from the Seed Residue of *Hippophae rhamnoides* L. Helv. Chim. Acta.

[28] Chand N., Naz S., Irfan M., Khan R.U., Rehman Z.U. (2018). Effect of Sea Buckthorn (*Hippophae rhamnoides* L.) Seed Supplementation on Egg Quality and Cholesterol of Rhode Island Laying Hens. Korean J. Food Sci. Anim. Resour..

[29] Krejcarová J., Straková E., Suchý P., Herzig I., Karásková K. (2015). Sea Buckthorn (*Hippophae rhamnoides* L.) as a Potential Source of Nutraceutics and Its Therapeutic Possibilities–A Review. Acta Vet. Brno..

[30] Cheng J., Kondo K., Suzuki Y., Ikeda Y., Meng X., Umemura K. (2003). Inhibitory Effects of Total Flavones of *Hippophae rhamnoides* L on Thrombosis in Mouse Femoral Artery and in Vitro Platelet Aggregation. Life Sci..

[31] Munekata P.E.S., Domínguez R., Pateiro M., Lorenzo J.M. (2020). Influence of Plasma Treatment on the Polyphenols of Food Products—A Review. Foods.

[32] Agbaria R., Gabarin A., Dahan A., Ben-Shabat S. (2015). Anticancer Activity of *Nigella sativa* (Black Seed) and Its Relationship with the Thermal Processing and Quinone Composition of the Seed. Drug Des. Devel. Ther..

[33] Ursache F.M., Ghinea I.O., Turturică M., Aprodu I., Râpeanu G., Stănciuc N. (2017). Phytochemicals Content and Antioxidant Properties of Sea Buckthorn (*Hippophae rhamnoides* L.) as Affected by Heat Treatment–Quantitative Spectroscopic and Kinetic Approaches. Food Chem..

